# Is muscle activation diverse in females with isolated patellofemoral osteoarthritis contrasted with age-matched healthy controls during stair descent task?

**DOI:** 10.3389/fphys.2024.1286406

**Published:** 2024-04-26

**Authors:** Jilan Adel, Reham E. Hamoda, Ayah Mahmoud Mohamed, Alaa Eldin Balbaa, Neama H. Neamat Allah, Hamada Ahmed Hamada

**Affiliations:** ^1^ Department of Physical Therapy for Musculoskeletal Disorders, Faculty of Physical Therapy, Kafrelsheikh University, Kafrelsheikh, Egypt; ^2^ Department of Physical Therapy for Woman’s Health, Faculty of Physical Therapy, Cairo University, Giza, Egypt; ^3^ Department of Physical Therapy for Obstetrics and Gynecology, Faculty of Physical Therapy, October 6 University, Giza, Egypt; ^4^ Basic Science Department, Faculty of Physical Therapy, Cairo University, Giza, Egypt; ^5^ Department of Physical Therapy for Musculoskeletal Disorders and its Surgery, Faculty of Physical Therapy, Cairo University, Giza, Egypt; ^6^ Dean of Faculty of Physical Therapy, Nahda University, Beni Suef, Egypt; ^7^ Department of Biomechanics, Faculty of Physical Therapy, Cairo University, Giza, Egypt

**Keywords:** patellofemoral osteoarthritis, EMG, muscular activation, gluteus medius, vastus medialis oblique, vastus lateralis, transversus abdominis, multifidus muscles

## Abstract

**Background:** Patellofemoral osteoarthritis (PF OA) is exceptionally predominant and limiting. However, little is known about the risk factors that contribute to its onset and progression.

**Purpose:** The aim of this study was to decide if women with PF OA descend stairs using different muscular activation strategies compared to similarly aged healthy controls.

**Methods:** Thirty-one women with isolated PF OA and 11 similarly aged healthy women took part in this study. The activation onset and duration of PF OA in vastus medialis oblique (VMO), vastus lateralis (VL), gluteus medius (GM), transversus abdominis (TrA), and multifidus muscles were evaluated during the stair descent task using surface electromyography (EMG).

**Results:** There was a non-significant difference between women with PF OA and healthy controls regarding all tested variables, except for the GM activation onset that was significantly delayed in women with PF OA, with the *p*-value of 0.011.

**Conclusion:** The causes of PF OA differ and might not always be due to a lack of quadriceps strength or VMO activation deficiency, and prospective longitudinal studies are required to confirm this assumption.

## Introduction

Patellofemoral osteoarthritis (PF OA) is the softening and damage of the cartilage lining under the kneecap (Eapan et al., 2011). PF OA is majorly found in the Middle East and Asia, while its rates of pervasiveness were found to be reduced in Estonia and Sweden. Approximately 39% of the subjects aged 30 years and above suffer from PF OA (Kobayashi et al., 2016).

Although PF OA is exceptionally predominant, limiting (Duncan et al., 2009; Duncan et al., 2011; Stoddart et al., 2021), and associated with impaired function (Hart et al., 2022), little is known about the risk factors that contribute to its onset and progression. It has been proposed that patellofemoral osteoarthritis might be a progression of patellofemoral pain syndrome (PFPS) (Duncan et al., 2011; Eijkenboom et al., 2018). Findings of recent studies have revealed an increased knee abduction during the sit-to-stand task (Hoglund et al., 2014) and hip adduction during gait (Crossley et al., 2018) in patients with PF OA compared with healthy controls. This increase in knee abduction and hip adduction might be the result of lower hip muscle activation or strength (Powers, 2010). In addition, a deficit in the activation or strength of the quadriceps may lead to patellar malalignment, which may significantly increase lateral retropatellar contact pressure due to decreased patellofemoral contact area (Powers et al., 2017).

Stair descent aggravates anterior knee pain (Briani et al., 2018) as it causes the highest patellofemoral load (Van Rossom et al., 2018). Most of the previous studies evaluated muscle activation in patients with PFPS (Brindle et al., 2003; Boling et al., 2006; Aminaka et al., 2011; Bolgla et al., 2011; Nakagawa et al., 2011; Rathleff et al., 2013; Motealleh et al., 2015; Yoosefinejad et al., 2022). Only two studies, to the best of the authors’ knowledge, compared muscle activation during a stair negotiation task in patients with PF OA to that in healthy controls, one during stair descent (Wyndow et al., 2019) and other during stair ascent (Adel et al., 2022). Because of the paucity of studies evaluating muscle activation in patients with PF OA, a recent meta-analysis stated that the proof of altered muscle activation in this cohort is of only extremely low certainty (Siqueira et al., 2022). Therefore, this study compared muscle activation strategies in PF OA patients to those in healthy controls.

## Materials and methods

### Design

This retrospective cross-sectional case-control study was carried out at the Biomedical Engineering Lab, Faculty of Engineering, Cairo University, from June 2022 to January 2023.

### Participants

Thirty-one female patients with PF OA were recruited from the Outpatient Clinic of Cairo University Hospitals [age: 44.7 ± 6.24 years; body mass index (BMI): 26.58 ± 5.01 kg/m2; duration of illness: 3.83 ± 1.82 years; and pain severity (11-point numerical rating scale): 6.9 ± 1.74], and 11 age-matched healthy controls were recruited through advertising in the surrounding community [age: 41 ± 6.292 years and BMI: 29.68 ± 3.6 kg/m2]. Before participating, all subjects were briefed on the study’s procedures and were required to sign a consent form. Before initiating this study, we obtained an ethical approval from the Institutional Review Board of the Faculty of Physical Therapy, Cairo University (No. P.T.REC/012/003437), and the study was then registered at clinicaltrials.gov (NCT05407077).

### Eligibility criteria

All participants were between 35 and 55 years of age (Geraci and Montagner, 2015). The inclusion criteria are as follows: anterior or retropatellar knee pain score greater than 3 on the 11-point numerical rating scale during at least two patellofemoral joint loading activities, such as stair ambulation, squatting, and/or standing up from a chair, and throughout the preceding month, this pain should have been experienced on most days (Crossley et al., 2016). Additionally, patients had radiographic proof of PF OA with a grade of less than 2 from posteroanterior views in accordance with the Kellgren–Lawrence (KL) osteoarthritis classification system to rule out the possibility of concurrent tibiofemoral arthritis (Hart et al., 2012). Patients were excluded if they met any of the following criteria: hip, lumbar spine, or foot pain that has persisted for more than 3 months or which required treatment; individuals who exercise for more than 2 hours per day or on alternate days; a history of fractures or surgeries in the lower extremities, spine, pelvis, hip, knee, or the foot; a hip or patellar subluxation or dislocation; an injury to the meniscus or any of the knee ligaments; fibromyalgia; neurological disorders; or systemic arthritis (Wyndow et al., 2019). Exclusion criteria specific for the healthy control group included any history of knee pain or pathology when engaging in any patellofemoral joint loading activities.

### Assessment

Before initiating the EMG measurements, information on demographic characteristics was obtained from all participants. All patients stated the duration of their illness, and the 11-point numerical rating scale (11-NRS) was used to measure the severity of pain.

### Surface electromyography

Muscle activation of gluteus medius (GM), vastus medialis oblique (VMO), vastus lateralis (VL), transversus abdominis (TrA), and multifidus muscles was recorded using an eight-channel high-resolution wireless bio-amplifier system (Biomation, Almonte, Canada) via bipolar Ag–AgCl disposable surface electrodes (better signal solution medical supply Co., Limited, Zhongshan, China). The recorded data were then sampled at 1,000 Hz and bandpass-filtered at 50–200 Hz and stored on a computer for further analysis using a custom program in MATLAB.

### Outcome measures

The data on the activation onset of the GM, VMO, VL, TrA, and multifidus muscles and the activation duration of the GM, VMO, VL, TrA, and multifidus muscles were collected from all participants.

### Procedures

The participant’s skin was prepared before electrode placement by shaving excess hair if needed and cleaned with alcohol to decrease skin impedance. Three disposable surface electrodes were used on each muscle. Two recording electrodes were placed over the muscle belly with an inter-electrode distance of around 30 mm (Park et al., 2010), while the third electrode was applied over the closest bony prominence.

The recording electrodes for the multifidus muscle were placed 2 cm lateral to the spinous process of the fifth lumbar vertebra (Park et al., 2010; Boudreau et al., 2011), while the recording electrodes for the transversus abdominis were placed 2 cm medial and inferior to the anterior superior iliac spine (ASIS) (Marshall and Murphy, 2003). For the GM, the recording electrodes were placed on one-third of the distance between the iliac crest and the greater trochanter (Park et al., 2010). For the VMO, the recording electrodes were placed 5 cm medial to a point that is one-fourth of the distance between the superior aspect of the patella and ASIS. The recording electrodes for VL were located halfway the distance between the lateral femoral epicondyle and the greater trochanter lateral to the rectus femoris (Kellis and Kouvelioti, 2009).

The stair descent task consisted of descending two steps (one foot on each step). The participants were instructed to stand on the second step and 5 cm away from its edge (Motealleh et al., 2015). Participants were then instructed to descend the steps barefoot with their arms hanging at the side of the body at their normal speed (Briani et al., 2016; Dorosti et al., 2017). Participants in the healthy control group were instructed to descend with the dominant limb (Saad et al., 2011), while participants in the PF OA group were asked to descend with their most painful limb (Motealleh et al., 2015).

Participants completed one practice stair descent attempt before data collection to become accustomed to the task. The participants then completed three test trials with a 30-s rest in between to avoid becoming fatigued. The mean of the three trials was used for analysis.

The recorded data were full-wave-rectified and high-pass-filtered at 75 KHz (fourth order). The baseline activity of each muscle when standing was measured 300 milliseconds before the start of each individual trial. Using MATLAB codes, each muscle was considered on when its activity exceeded the threshold of 3 SDs (standard deviations) above its baseline level and stayed there for a minimum of 25 msec (Cowan et al., 2001). On the other hand, the muscle was considered off when its activity decreased below the threshold of 3 SDs for at least 25 msec, and the duration of activation of each muscle was defined as the time between its activation onset and offset (Aminaka et al., 2019).

### Sample size estimation

**FIGURE 1:**
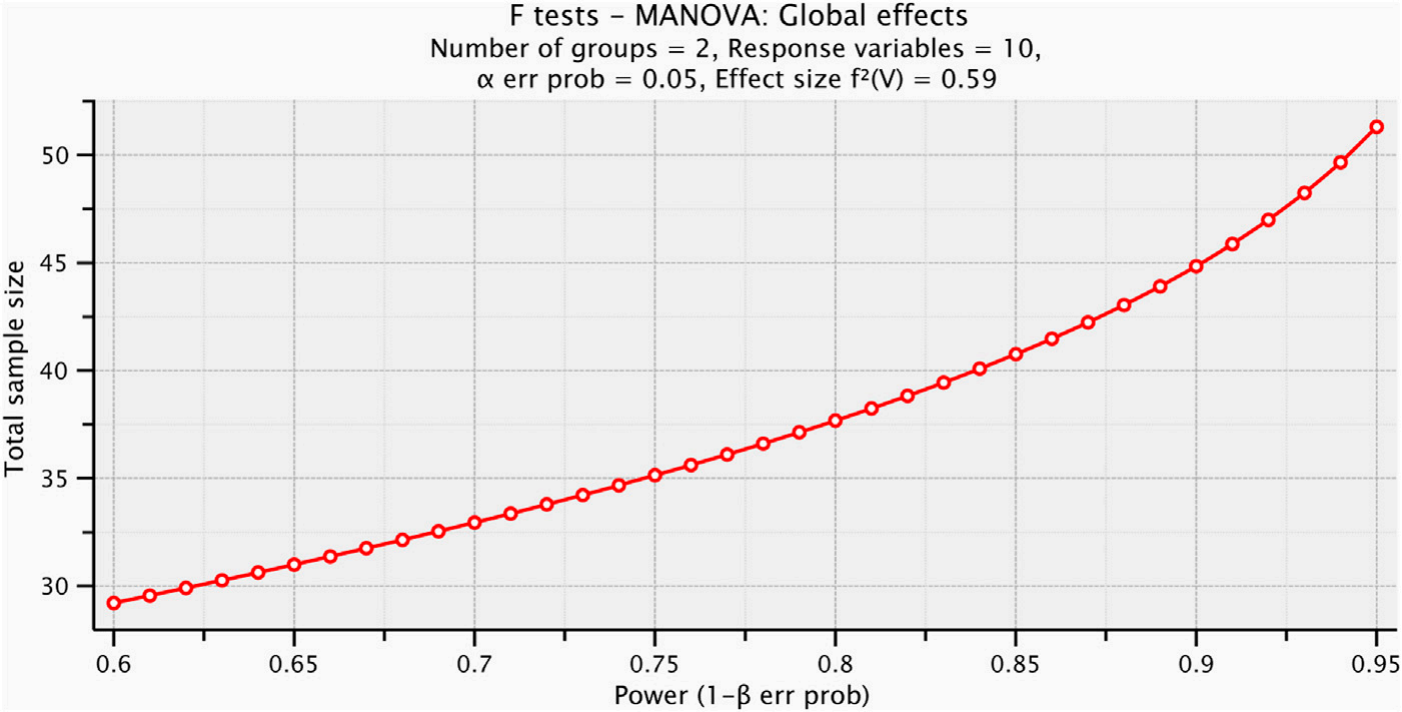
Plot of sample size calculation.

Before starting the test procedures, a pilot study was conducted with ten participants to determine the appropriate sample size. Test size estimation was performed preceding the investigation utilizing G*POWER statistical programming (version 3.1.9.2; Franz Faul, Universitat Kiel, Germany) [F tests: MANOVA: global effects, α = 0.05, β = 0.2, power = 80%, partial η2 = 0.372, and effect size = 059], and it was revealed that the appropriate sample size for this study was N = 38, as shown in Figure 1. We collected a larger number of participants than the calculated sample size to overcome missing of participants, which might occur.

### Statistical analysis

All statistical analyses were conducted using Statistical Package for Social Sciences (SPSS) for Windows, version 23 (SPSS, Inc., Chicago, IL). This study involved ten dependent variables, which are the activation onset and duration of the VMO, VL, GM, TrA, and multifidus muscles, while the tested group had only independent variable, and it had two levels: the healthy control group and the PF OA group. The one-way between-subject multivariate analysis of variance (MANOVA) test was performed to compare the mean values of the independent variables in PF OA women to similarly aged healthy controls. An independent *t*-test was used to compare the baseline demographics (age and BMI) between groups. The significance level was set at *p* < 0.05.

## Results

### Baseline demographics of the included participants

The results yielded a non-significant difference in the mean values of all ages and BMI between age-matched healthy controls and participants with PF OA as the probability level was above the significance level set for this study (Table 1).

**TABLE 1:** Baseline demographics data of the included participants.

	Healthy (*n* = 11)	PF OA (*n* = 31)	*p*-value
	X ± SD	X ± SD	
**Age (years)**	41 ± 6.292	44.7 ± 6.24	0.099
**BMI (kg/m** ^**2**^ **)**	29.68 ± 3.6	26.58 ± 5.01	0.075
**Illness duration (years)**	-	3.83 ± 1.82	
**Pain severity (11-NRS)**	-	6.9 ± 1.74	

Data were displayed as mean ± standard deviation (SD), PF OA = patellofemoral osteoarthritis; BMI, body mass index; NRS, numerical rating scale, *p*-value = probability value, the significance level was set at *p* < 0.05.

### The activation onset and duration of the tested muscles

One-way between-subject MANOVA yielded a non-significant difference in the mean values of the activation onset and duration of the tested muscles (F = 1.44, *p* = 0.211, partial η^2^ = 0.59). The univariate tests of one-way between-subject MANOVA demonstrated a non-significant difference in the mean values of the activation duration of the five tested muscles between both groups (*p* > 0.05) (Table 2). In the same context, there was no significant difference in the mean values of the activation onset of TrA, VMO, VL, and multifidus muscles (*p* > 0.05). On the other hand, the univariate tests revealed a significant difference in the mean values of the activation onset of GM between both groups as the probability level was 0.011. Subsequently, *post hoc* tests used for multiple pairwise comparison yielded a significant increase in the mean value of GM activation onset in favor of female patients with PF OA (*p* = 0.011) (Table 3).

**TABLE 2:** Activation duration of the tested muscles.

Muscle	Healthy (*n* = 11)	PF OA (*n* = 31)	MD	F- value	*p*-value	Cohens effect size
**VMO**	0.35 ± 0.21	0.29 ± 0.19	0.06	0.693	0.41	0.2857
**VL**	0.23 ± 0.12	0.24 ± 0.2	−0.01	0.04	0.842	0.083
**GM**	0.27 ± 0.15	0.34 ± 0.2	−0.07	0.661	0.421	0.4667
**Multifidus**	0.14 ± 0.13	0.25 ± 0.2	−0.11	2.61	0.114	0.8461
**TrA**	0.22 ± 0.07	0.23 ± 0.2	−0.01	0.006	0.94	0.1429

**TABLE 3:** Activation onset of the tested muscles.

Muscle	Healthy (*n* = 11)	PF OA (*n* = 31)	MD	F- value	*p*-value	Cohens effect size
**VMO**	1.47 ± 0.55	1.78 ± 0.54	−0.31	2.675	0.11	0.5636
**VL**	1.27 ± 0.56	1.38 ± 0.96	−0.11	0.134	0.717	0.1964
**GM**	1.38 ± 0.47	1.95 ± 0.64	−0.57	7.223	0.011*	1.2127
**Multifidus**	1.2 ± 0.66	1.71 ± 0.78	−0.51	3.659	0.063	0.7727
**TrA**	1.53 ± 0.42	1.44 ± 0.67	0.09	0.158	0.693	0.2143

Data were displayed as mean ± standard deviation, VMO, vastus medialis oblique; VL, vastus lateralis; GM, gluteus medius; TrA, transversus abdominis; MD, mean difference, *p*-value, probability value, *the significance level was set at *p* < 0.05.

## Discussion

This study aimed to investigate the activation onset and duration of transversus abdominis (TrA), multifidus, gluteus medius (GM), vastus medialis oblique (VMO), and vastus lateralis (VA) in patients with isolated patellofemoral osteoarthritis (PF OA) during a stair descent task. This study found that female patients with PF OA had significantly delayed activation of GM during stair descent when compared to healthy controls. Other tested variables, on the other hand, showed no significant difference between the two groups.

Regarding VMO activation onset, this study demonstrated that PF OA female patients and their healthy counterparts had a non-significant difference, and this result corroborated the previous findings of Brindle et al. (2003), Aminaka et al. (2011), Bolgla et al. (2011), Rathleff et al. (2013), Wyndow et al. (2019), and Yoosefinejad et al. (2022). On the other hand, this non-significant difference is comparable to the findings of Motealleh et al. (2015) as their results showed a delayed onset of VMO in male patients with PFP, and the reasons behind these comparable results were the heterogeneity of recruited subjects, muscle onset determination, electrode placement, and speed of movement. Regarding electrode placement, Motealleh et al. (2015) placed VMO electrodes 4 cm superior to and 3 cm medial to the superomedial patellar margin and oriented them 55^°^ to the vertical. Thus, they did not consider the subjects’ height despite its importance as it could vary significantly; therefore, a percentage of limb length should be considered to assure electrode placement standardization for individuals and improve the between-subject and between-study comparisons (Wong and Ng, 2006). In addition, the participants in this study were asked to descend the stairs at their normal speed, while Motealleh et al. (2015) instructed their participants to step down the stairs at their fastest pace. As long as movement at a higher speed might affect the electrical activity of muscles, especially when the movement is painful, movement speed could be another source of heterogeneity between studies (Dorosti et al., 2017).

For the onset of activation of the VL, the results of the current study indicated that there was no significant difference between female patients with PF OA and healthy subjects. This finding is in line with the previous findings of Brindle et al. (2003), Bolgla et al. (2011), Rathleff et al. (2013), and Yoosefinejad et al. (2022). Contrary to our findings, Motealleh et al. (2015) reported that the VL was activated earlier in subjects with PFP than in the healthy controls. Adding to the previously mentioned factors that might have led to this contrast, Motealleh et al. (2015) did not consider the patient’s height when applying electrodes on the VL same as VMO, unlike in this study, despite its necessity.

In the same context, Wyndow et al. (2019) disagreed with our findings as they documented an earlier activation of the VL during stair descent in patients with PF OA compared to healthy subjects. This discrepancy could be explained by different characteristics of the recruited subjects. Although they concurred with this study in recruiting patients with PF OA and not PFP, as in most of the previous works, there was some dissimilarity. They included patients from both sexes despite the positive association between gender differences and EMG activity (Hughes and Dally, 2015; Peng et al., 2020). When evaluating gait and clinical parameters in PF OA patients, studies should take sex variations into account (Hart et al., 2024). Moreover, they allowed patients with mild tibiofemoral arthritis to take part in their study.

Regarding the duration of VMO activation, the findings of this study demonstrated that there was no statistically significant difference between either of the groups. Thus, our results agreed with the previous findings of Aminaka et al. (2011) and Wyndow et al. (2019) but opposed to the findings of Brindle et al. (2003), whose results yielded a shorter duration of VMO activity during stair descent in male and female patients with PFP compared to controls, and this contradiction may be due to different characteristics of the recruited subjects, including age and gender. Moreover, they determined muscle onset and offset in a different method from the current study by adjusting the amplitude threshold to 5 standard deviations above baseline noise rather than 3 standard deviations, as in this study.

This study demonstrated that the activity duration of VL in female patients with PF OA was not significantly different from that in controls when descending stairs. On the contrary to this finding, Brindle et al. (2003) reported a shorter duration of VL activation in subjects with PFP as opposed to controls. In addition, Wyndow et al. (2019) found that the duration of VL activation was longer in patients with PF OA than in controls during stair descent.

Regarding onset of GM activation, the results of this study revealed that onset of GM activation was significantly delayed in PF OA female patients as opposed to their healthy counterparts. This finding is in line with the previous findings of Aminaka et al. (2011) and Motealleh et al. (2015). A recent study indicated that patients with isolated PF OA displayed significantly increased hip adduction at 30, 45, and 60 degrees of knee flexion during the descent phase of single-leg squat (Carvalho et al., 2024), which could be a speculative reason for the delayed onset of GM activation. Moreover, while the delayed onset of GM activation may have a role in the etiology of patellofemoral osteoarthritis, it is also possible that PF OA patients experience this delayed onset due to lowered activity as a pain-reduction strategy.

On the other hand, Brindle et al. (2003), Boling et al. (2006), Bolgla et al. (2011), and Nakagawa et al. (2011) disagreed with our results as they found a non-significant difference between subjects with PFP and healthy ones with respect to GM activation onset during stair descent. These conflicting results reflected differences in the characteristics of the recruited subjects. Adding to the different sample profile, Boling et al. (2006), Bolgla et al. (2011), and Nakagawa et al. (2011) controlled the timing of stair-stepping. On the other hand, subjects in our study descended the stairs at their normal speed. We did not control the speed of stair negotiation because, as previously demonstrated for gait in asymptomatic subjects, controlling the timing of stair negotiation can alter the electromyographic signals (Jordan et al., 2007). Moreover, Nakagawa et al. (2011) determined muscle activation onset using an amplitude threshold of 2 SDs instead of 3 SDs of baseline noise, which might have contributed to the disagreement between their results and ours. In the same context, Wyndow et al. (2019) contradicted our results as they demonstrated that EMG onset of GM in females with PF OA was not significantly different from that in controls during stair descent.

Regarding the GM activation duration, the results of this study revealed a non-significant difference between either of the groups, and this finding agreed with the previous findings of Boling et al. (2006) and Wyndow et al. (2019). On the contrary to this finding, Brindle et al. (2003) and Aminaka et al. (2011) found that individuals with PFP demonstrated a shorter duration of GM activity in contrast with the healthy subjects during stair descent.

Only one study, to the best of the authors’ knowledge, evaluated the activation onset of the transversus abdominis in patients with PFP as they descended stairs, and the results showed a delayed activation of TrA compared to that in controls (Motealleh et al., 2015). Hence, this finding disagreed with those of the current study, which revealed a non-significant difference between either of the groups: isolated PF OA female patients and healthy controls.

Regarding the activation onset and duration of multifidus muscle activation, not to mention the duration of transversus abdominis activation, the current results revealed a non-significant difference between female patients with PF OA and controls. Only one study, to the best of the authors’ knowledge, compared those variables between patients with PF OA and controls, but during stair ascent, which is different from the task used in this study, and revealed a delayed activation of multifidus muscles in the PF OA group (Adel et al., 2022). Hence, we have no studies to compare our results with. Previous studies found non-significant differences between subjects with PF OA and controls regarding trunk kinematics on the sagittal plane during stair negotiation (Fok et al., 2013) and the descent phase of single-leg squat (Carvalho et al., 2024). This might explain why multifidus and TrA activation onset and duration are not significantly different between both groups, but prospective longitudinal studies are essential to ascertain this assumption.

Some noteworthy limitations that might have accounted for the non-significant results in this study should be noted: first, we included patients in this study if they had a minimum pain chronicity of 1 month, but we did not determine any upper limits for pain chronicity. Moreover, recent literature addressed the presence of kinesiophobia (fear of re-injury due to movement) in patients with anterior knee pain (Maclachlan et al., 2017; Smith et al., 2018; Priore et al., 2019; Maclachlan et al., 2020). In women with PFP, there was a strong correlation between protective kinematic impairments, such as decreased peak knee flexion and cadence and kinesiophobia, but not with knee extensor strength during a stair descent task. Furthermore, no correlation was revealed between kinesiophobia and knee extensor strength. Thus, kinesiophobia may have a greater impact on mobility deficits than strength (Silva et al., 2019), and its reduction might have a positive effect on anterior knee pain (Doménech et al., 2014). Put together, kinesiophobia might have affected the recruitment strategies of our patients, and future studies are necessary to confirm this assumption.

## Conclusion

The findings of this study revealed that EMG activity of the GM was substantially delayed in female subjects with PF OA during stair descent when compared to healthy controls. However, because the cross-sectional study design did not allow us to detect whether this delayed activity was an underlying cause for the development of PF OA or a result of PF OA, these findings should be regarded cautiously. Prospective longitudinal studies are thus required.

## Clinical implications

• The causes of PF OA differ and might not always be due to a lack of quadriceps strength or VMO activation deficiency.
• Before recommending a treatment strategy, the activation and strength of the gluteus medius and quadriceps muscles should be examined.

## Data Availability

The raw data supporting the conclusion of this article will be made available by the authors, without undue reservation.
